# Book Notices and Reviews

**Published:** 1892-05

**Authors:** 


### Book Notices.

R. C. M. PAGE, M. D. Author of "A Chart of Physical signs of Diseases of the Chest," "A Hand-book of Physical Diagnosis of Diseases of the Organs of Respiration and Heart;" Professor of General Medicine and Diseases of the Chest in the New York Polyclinic; Visiting Physician to Randall's Island Hospital, and the Northwestern Dispensary, Department of Diseases of the Heart and Lungs; Member of the New York Academy of Medicine, and New York Pathological Society.

A text-book of the practice of medicine for the use of students and practitioners. Octavo, 578 pages, illustrated. Red parchment muslin. Price, $4.00. Published by Wm. Wood & Co., New York.

TREATISE ON MEDICAL AND SURGICAL GYNÆCOLOGY. By S. Pozzi, M. D., Professeur Agrége á la Faculté de Mèdicine, Chirurgien de l'Hopital Lourcine-Pascal, Paris. Complete in two volumes. Translated from the French Edition under the supervision of, and with additions by, Brooks H. Wells, M. D., Lecturer on Gynæcology at the New York Polyclinic; Fellow of the New York Obstetrical Society, and the New York Academy of Medicine. Volume Two. With 174 Wood engravings and 9 Full-page Plates in Color. Royal Octavo. 174 Wood-cuts. Muslin, $6.00; sheep, $7.00; half morocco, $8.00.

There has been a growing desire for some work which should give, in a condensed and, at the same time, a practical form, what is best and most important in the various works on Gynæcology, not only in Europe, but in America. This has been accomplished by the author, Prof. S. Pozzi, in the present work.

This is not an exposition of any one system or school, nor are the methods presented those of any one man, or those of any one nationality; but the work is indeed a most complete digest of the best and most important writings on Gynæcology in France, Germany, England, and America.

From the fact that it is an epitome of the best literature of the subject to date, and includes the valuable additions of Dr. Brooks H. Wells, who has thoroughly adapted the work to the practice of the profession in America, it is an invaluable and necessary book.

---

A PRACTICAL TREATISE ON DISEASES OF THE SKIN. By Henry G. Piffard, A. M., M. D., Clinical Professor of Dermatology, University of the City of New York; Surgeon in Charge of the New York Dispensary for Diseases of the Skin; Consulting Surgeon to Charity Hospital; Consulting Surgeon to the Bureau of Out-door Relief, Bellevue Hospital; Consulting Dermatologist to the Board of Health, etc. With Fifty full-page Original Plates and Thirty-three Illustrations in the Text.

We think we can safely say this is the best work on skin diseases for the general practitioner we have yet seen. The plates are excellent, and a great help to diagnosis. What we like about the book is, the author tells one just what he wants to know; i. e., 1st, how to make a diagnosis, and 2nd, what to do to cure the case. There is no discussion and comparisons of methods, but the writer just states what medicines he has found best suited for such and such a condition, and how best to administer or apply them. This is a book of practical value.

---

ESSENTIALS OF PHYSIOLOGY. Arranged in the Form of Questions and Answers; Prepared especially for Students of Medicine. By H. A. Hare, B. Sc., M. D. Third Edition. Thoroughly revised and enlarged. 1891. Pages, 193. Price, $1. Send to Publisher, W. B. Saunders, 913 Walnut St., Philadelphia.

---

ESSENTIALS OF NERVOUS DISEASES AND INSANITY, their Symptoms and Treatment. A Manual for students and practitioners. By John C. Shaw, M. D., of Long Island Medical College. 1892. Pages, 194. Price, $1.00. Publisher, W. B. Saunders, 913 Walnut St., Philadelphia.

---

A MANUAL OF HYPODERMIC MEDICATION; The Treatment of Diseases by the Hypodermatic or Subcutaneous Method. By Roberts Bartholomew, A. M., M. D., LL. D., Philadelphia. Fifth Edition, Revised and Enlarged. 1891. Pages, 540. Price, Cloth, $3.00. Philadelphia: J. B. Lippincott Company. This is one of the first works ever written on hypodermic medication, and it can be said also that it is one of the *best*. It enjoys the title of *standard*.

---

SYLLABUS OF THE OBSTETRICAL LECTURES in the Medical Department of the University of Pennsylvania. By R. C. Norris, A. M., M. D. Second Edition. 1891. Pages, 198. Price, $2.00. Philadelphia: W. B. Saunders, 913 Walnut St.

---

DSEASES OF THE NERVOUS SYSTEM. By Jerome K. Bauduy, M. D., LL. D., Professor of Diseases of the Nervous System and the Mind in Missouri Medical College, St. Louis. Second Edition. 1892. Price, —. Pages, 352. Philadelphia: J. B. Lippincott Company.

---

THE PRINCIPLES AND PRACTICE OF MEDICINE. Designed for the use of Practioners and Students of Medicine. By William Osler, M. D., Fellow of the Royal College of Physicians, London; Professor of Medicine in the Johns-Hopkins University, and Physician in Chief to the Johns-Hopkins Hospital, Baltimore. Formerly Professor of the Institute of Medicine, McGill University, Montreal, and Professor of Clinical Medicine in the University of Pennsylvania, Philadelphia. New York: D. Appleton & Co., Publishers. Price: cloth, $5.50; sheep, $6.50; half morocco, $7.00. pp. 1079.

This book is well printed, well written, and its arrangement and classification of subject matter is all that could be expected, coming from a man of such vast experience as a clinician, teacher and author as Dr. Osler. It is divided into eleven sections, briefly as follows: Section I, Specific Infection; Section II, Constitutional Diseases; Section III, Diseases of the Digestive System; Section IV, Diseases of the Respiratory System; Section V, Diseases of the Circulatory System; Section VI, Diseases of the Blood and Ductless Glands; Section VII, Diseases of the Kidneys; Section VIII, Diseases of the Nervous System; Section IX, Diseases of the Muscles; Section X, The Intoxications, Sun-stroke, Obesity; Section XI, Diseases due to Animal Parasites.

This book is eminently practical, and is well abreast of the various advances in medical science. The chapter on dengue fever, in which reference is made to the bacterium discovered in the blood of patients suffering from this disease by Dr. J. W. McLaughlin, of Austin, does not properly classify the micro-organism. Dr. Osler refers to it as a streptococcus, when in fact it is an unclassified coccus which differs in its group-forms from any pathogenic bacterium heretofore described. B.

---

AGE OF THE DOMESTIC ANIMALS, being a complete treatise of the dentition of the horse, ox, sheep, hog and dog, by Rush Shippen Huidekoper, M. D., of the Veterinary College, N. Y., 200 illustrations. 1891. Pp. 225. Price, $1.75 postpaid. Published by F. A. Davis Co., 1231 Filbert St., Philadelphia.

---

THE PHYSICIAN AS A BUSINESS MAN; how to obtain the best financial results in the practice of medicine. By J. J. Taylor, M. D. 1891. Price ——. Pp. 144. Published by the Medical World, 1520 Chestnut street, Philadelphia.

---

SAUNDERS' POCKET MEDICAL FORMULARY. By William M. Powell, M. D., of Philadelphia. 1891. Pp. 260. Price, cloth, $1.50. Published by W. B. Saunders, 913 Walnut St., Philadelphia.

---

3000 QUESTIONS ON MEDICAL SUBJECTS—Arranged for self-examination; sent to any medical student on receipt of 10 cts. by Publishers, P. Blakiston, Son & Co., 1012 Walnut Street, Philadelphia.

---

TABLES FOR DOCTOR AND DRUGGIST, comprising: I. Table of Solubilities; II. Table of Reactions and Incompatibles; III. Table of Doses and Uses of Medicines; IV. Table of Specific Gravities; V. Table of Poisons and Antidotes. Compiled by Eli H. Long, M. D., Buffalo College of Pharmacy. 1891. Price, —. Geo. S. Davis, Publisher, Detroit.

---

PULMONARY CONSUMPTION AS A NERVOUS DISEASE. By Thos. J. Mays, M. D., Philadelphia. Pages, 184. Price, 25 cents. 1891. Geo. S. Davis, Publisher, Detroit.

---

HISTORY OF CIRCUMCISION, from the earliest times to the present. Moral and physical reasons for its performance, with a history of eunuchism, hermaphrodism, etc., and of the different operations practiced upon the prepuce. By P. C. Remondino, M. D., San Diego, California. 1891. Price, $1.25. Pages, 346. Publisher: F. A. Davis, Philadelphia.

A, B, C, OF THE SWEDISH SYSTEM OF EDUCATIONAL GYNASTICS. A practical hand-book for schools, teachers and home. By Hartvig Nissen, Boston. 1891. Price, 75 cents. Pages, 107. F. A. Davis, Publisher, Philadelphia.

